# Bilateral Breast Phyllodes Tumor in Androgen Insensitivity Syndrome

**DOI:** 10.1055/s-0042-1758668

**Published:** 2023-02-03

**Authors:** Aishwarya Sunil Dutt, Girish Bakhshi, Chandrakant Sabale, Ravi Landge, Sushrut Baligar, Rajalakshmi V., Madhu Jha, Sampada Joshi, Chettubattina Ravi Teja

**Affiliations:** 1 Department of General Surgery, Grant Government Medical College and Sir J. J. Group of Hospitals, Mumbai, Maharashtra, India

**Keywords:** phyllodes, complete androgen insensitivity syndrome, testis

## Abstract

Phyllodes is a rare tumor found exclusively in females. It can be classified into benign, intermediate, or malignant variety based on the aggressive nature of the disease. With adequate preoperative clinical assessment combined with histopathology and radiological investigations the adequate treatment strategy can be formulated to avoid future recurrences. Complete androgen insensitivity syndrome (CAIS) is associated with a genotypic male, which can be confirmed by karyotyping, with phenotypic female characteristics. The present case is the first case of bilateral breast phyllodes tumor in a patient with CAIS. Preoperative assessment was suggestive of bilateral phyllodes tumor with bilateral gonads in the inguinal region which was confirmed to be testis postoperatively on histopathological analysis. A brief case report with review of literature is presented.

Androgen insensitivity syndrome (AIS) is a disorder resulting from complete or partial resistance to the biological actions of androgens in an XY karyotype causing a female phenotype with normal testis determination and production of age-appropriate androgen concentrations. 1 Partial AIS (PAIS) has phenotype of varying degrees of masculinization which could even be mild intensity such as gynecomastia or infertility in healthy men. They may present with ambiguous genitalia, microphallus, severe hypospadias, and bifid scrotum that may contain gonads. 2 Complete AIS (CAIS) on the other hand has a complete female phenotype with testis and male equivalent androgen levels. They present with normal pubertal growth spurt and breast development with history of primary amenorrhea.

AIS has been associated with gonadal malignancies, namely germ cell tumors. These are more commonly seen in PAIS with a 6 to 15% incidence or higher if the testis are present intra-abdominally. 1 Other tumors such as breast tumors are uncommon and are related to excessive hormone administration. 2

Phyllodes tumor is considered a rare tumor found in females. Although the exact etiology for the tumor is not certain, it has a familial association and is associated with syndromes such as Li–Fraumeni. The patients present with age ranging between 9 and 93 years. 3 There have not been any documented cases of phyllodes in association with AIS till date. This is a case report of a 60-year-old patient with CAIS presenting with bilateral phyllodes tumor of the breasts.

## Case Report

A 60-year-old female presented with a bilateral breast lump since 3 years. The two lumps grew in size with the left breast lump being larger than the right and both involving the entire breast with a wound over the upper and outer aspect of the left breast. On further enquiry, patient gave history of amenorrhea since childhood and did not bare any children.

On examination, she was found to have a lump in both breasts which involved all four quadrants. The left breast lump was larger and involved the skin. Both lumps were firm and bosselated with dilated superficial veins. An ulcer of 2 × 2 cm was present in the upper and outer quadrant of the left breast which was healthy. Central group of lymph nodes were palpable in the left axilla which were firm with restricted mobility. Right side lump was not attached to the underlying chest wall with no dilated veins and no axillary lymph node involvement.

**Fig. 1:**
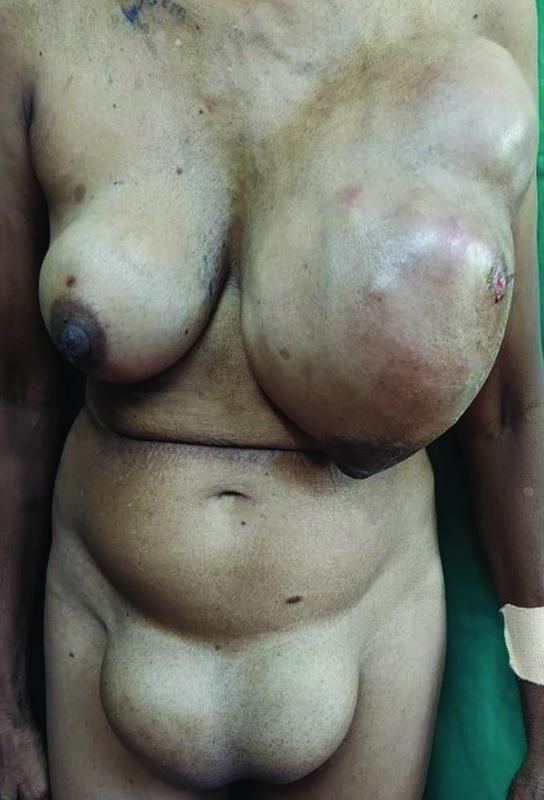
Anterior profile of the clinical presentation with bilateral breasts lumps (right more than left) and bilateral inguinal masses.

She was found to have bilateral swellings in the inguinal region which she claimed to have since childhood. The swellings were both 10 × 6 cm in size and directed inferomedially ( Fig. 1 ). They were firm, mobile, and increased in size on coughing.

Perineal examination showed feminine characteristics with a clitoris and labia minora and majora. Vaginal vault prolapse was visible. On per vaginal examination the cervix and uterus were not palpable. Per rectal examination was unremarkable.

Ultrasound (USG) of the breasts showed ill-defined solid masses with cystic spaces involving all quadrants in the left side and the upper outer and inner quadrants of the right breast. A left axillary lesion was also present. Internal vascularity was seen with no evidence of calcifications within suggestive of sarcomatous changes likely phyllodes. USG abdomen and pelvis showed agenesis of the uterus with right and left inguinal masses with few cystic areas and microcalcifications.

Core needle biopsy of bilateral breast lumps was taken. Right breast biopsy showed fragments of stromal tissue with scanty foci of papillary lesions and no evidence of malignancy. Left breast biopsy showed fragments of stromal tissue with scanty foci of papillary lesion. On immunohistochemistry, the left breast biopsy tissue showed the myoepithelial cells highlighted within and around the lesion with p40 and calponin. This was suspicious for papillary neoplasm.

**Fig. 2:**
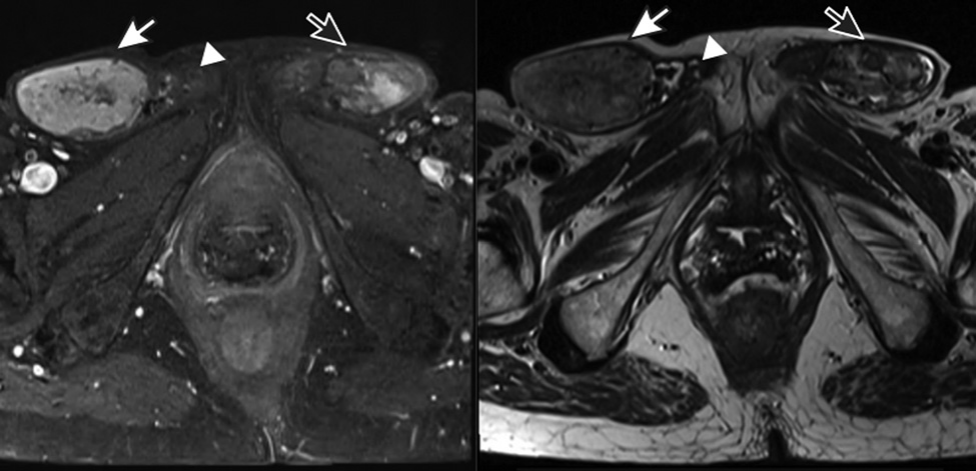
T1- and T2-weighted magnetic resonance imaging (MRI) transverse section of pelvis showing bilateral testis. White arrow showing the right testis, white arrow head showing the right epididymis, and the black arrow with white outline showing the left testis.

Magnetic resonance imaging (MRI) of the pelvis was done to further investigate the herniated mass. It showed bilateral inguinal heterogeneous structures measuring 3.2 × 5.2 cm on the right and 4.3 × 2.6 cm on the left. The lesion showed heterogeneous hyperintensity on T2-weighted image (T2W1), isointense on T1WI, and heterogeneous postcontrast enhancement ( Fig. 2 ). There was mild fluid surrounding both the masses. The findings were suggestive of bilateral testis with malignant transformation. There were also structures resembling epididymis and spermatic cords. Bilateral patent processus vaginalis was visualized through which herniation of inguinal masses with small bowel was visible. There was a presence of vagina with an absence of Mullerian duct structures and ovaries. Posterior urethrocele and anorectal descent below the level of pubococcygeal line was seen in neutral position. Bilateral lobulated breast masses (left > right) were visible with left breast mass being more heterogeneous and with indistinct fat planes with the left chest wall.

A positron emission tomography with computed tomography scan was done which showed massive metabolically active mildly lobulated mass lesion in the left breast parenchyma (with deep component showing high grade metabolically activity) representing a neoplastic pathology which favored sarcomatoid pathology. A similar multilobulated heterogeneous mass lesion in the right breast showed low-grade metabolic activity. Herniation was seen in bilateral labia with bowel loops and omental fat as its contents. There was no active disease elsewhere.

**Fig. 3:**
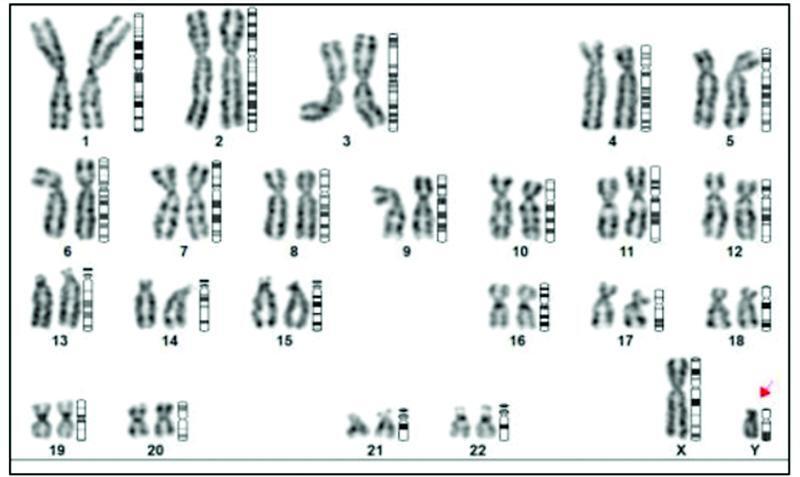
Peripheral venous blood karyotyping showing 46XY disorder of sex differentiation.

Karyotyping was done which showed 46XY disorder of sex differentiation ( Fig. 3 ). Chromosome analysis revealed genetic sex complement to be that of male with no evidence of any structural or numerical abnormality. Hence, there was discordance between the chromosomal, gonadal, and phenotypic sex.

Hormone study was done which showed a follicular-stimulating hormone (FSH) of 6.86 mIU/mL, testosterone of 5.27 ng/mL (correlating to male), anti-Mullerian hormone of 2.395 ng/mL, serum dehydroepiandrosterone sulfate (DHEA SO4) of 184.2 (correlating to male), and luteinizing hormone (LH) of 4.967 mIU/mL. Tumor markers showed an alpha-fetoprotein value of 1.55 IU/mL, CA-125 of 39.91 U/mL, CEA of 3.39 ng/mL, and beta-human chorionic gonadotropin (beta-HCG) of 4.43 mIU/mL (raised for males).

The overall clinico-radiological features were suggestive of a CAIS with female phenotype (breast tissue formation with lump) and male genotype (presence of testis, epididymis, and spermatic cords). Further investigations also confirmed the presence of raised male hormones with a raised beta-HCG tumor marker.

The operative decision was taken to do a left modified radical mastectomy (MRM) due to the aggressive nature and involvement of the left axillary lymph nodes, suggestive of a malignant variety of phyllodes. This was planned alongside a right wide local excision along with a bilateral gonadectomy and hernia repair.

Intraoperatively, the breast masses were seen to be highly vascular. Left MRM and right side wide local excision was done. Closure was done with simple polyamide sutures with drains placed bilaterally. Bilateral gonads were also dissected and seen to rest in the inguinal canals from where they were carefully dissected out and a high inguinal ligation of the cord with vessels was done. Bilateral hernioplasty was done.

Grossly, the right breast mass was 11 × 10 × 5 cm, lobulated, and firm. It was well encapsulated. On cutting open it was seen to be grayish white and lobulated with no areas of necrosis or hemorrhage. The left breast mass with axilla measured 32 × 19 × 11 cm with the tumor reaching up to the skin. It was lobulated with a variegated appearance with solid, firm to hard areas, with areas of hemorrhage and areas of myxoid changes. Twelve lymph nodes were excised with the largest lymph node being 2 × 2 × 1 cm in size.

The right gonad consisted of a testis of 7 × 6 × 4 cm with a spermatic cord of 7 cm. It was encapsulated, smooth, and consisted of congested vessels. On cut section there were multiple yellowish white nodules with areas of fibrosis and no necrosis or hemorrhage identified. The left gonad consisted of a testis measuring 6 × 4 × 2.5 cm and a spermatic cord of 8 cm. It was encapsulated and smooth with congested blood vessels. Cut section showed multiple yellowish white nodules with areas of fibrosis and no necrosis or hemorrhage.

**Fig. 4:**
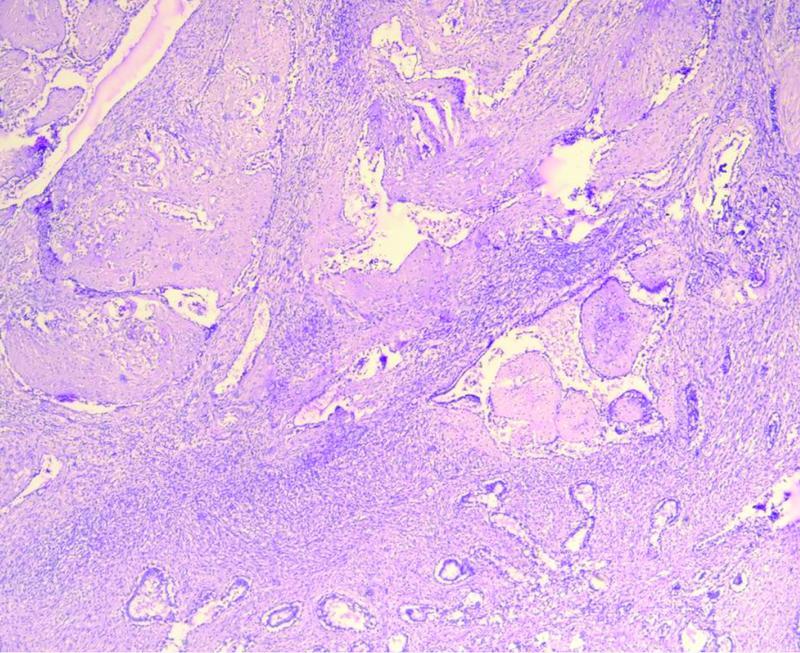
Low power view of the right breast showing benign phyllodes with overgrown stroma and sparse epithelial elements.

**Fig. 5:**
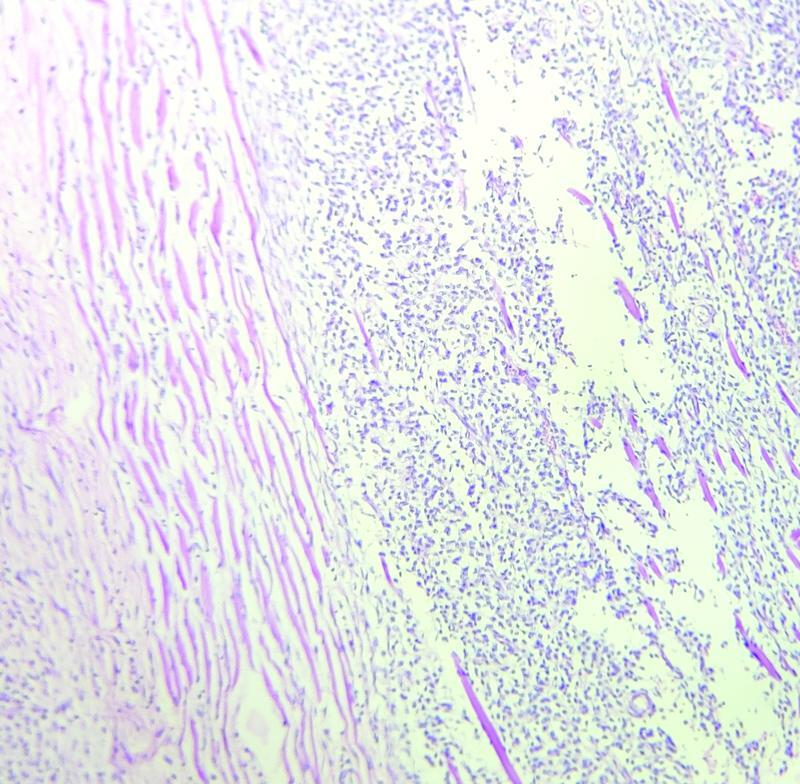
Low power view of the left breast showing malignant phyllodes with muscle invasion.

Microscopic examination of the right breast tissue was suggestive of benign phyllodes tumor with free margins ( Fig. 4 ). Left breast tissue and lymph nodes were suggestive of malignant phyllodes tumor with fibrosarcomatous, osseous, and chondroid areas seen along with muscle invasion ( Fig. 5 ). Margins of the specimen were free of tumor. Bilateral gonads showed Leydig cell hyperplasia with Reinke crystals and wit bilateral spermatic cord free of tumor.

**Fig. 6:**
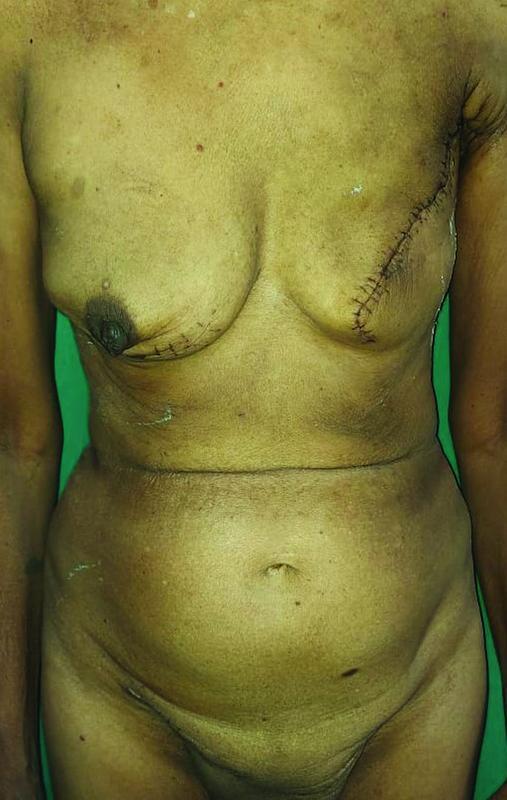
Anterior profile of the postoperative presentation prior to discharge.

Patient was discharged on postoperative day 8 after all subcutaneous drains were removed with a healthy suture line ( Fig. 6 ). After suture removal, patient was advised to undergo adjuvant radiotherapy in view of malignant phyllodes of the left breast. Follow-up of 9 months has shown the patient to be symptom and disease free.

## Discussion

AIS is a 46XY disorder of sexual development caused by inactivation of or deletion in the X-linked androgen receptor (AR) gene. 4 This leads to decrease or abolishment of the androgenic effects on target tissues depending on the degree of responsiveness of the genitals to external androgens. It is caused by a mutation of the AR gene at Xq11Y12. 5 AIS is traditionally divided into three general phenotypical subgroups: CAIS with female genitalia; PAIS with predominantly female, male, or ambiguous genitalia; and mild AIS with male genitalia. 6

Development of estrogen-dependent secondary sexual characteristics occurs as the result of excess aromatization of androgens. 1 They could present with breast development, amenorrhea, lack of axillary and pubic hair, absence of uterus and fallopian tubes, gonads in the abdomen, inguinal region or labia, and have a taller physique. All these features were present in the present case. Inguinal hernias are seen with AIS with 80 to 90% of those with CAIS 5 as in the present case. Endocrine features in AIS show an endocrine profile of a hormone-resistant state with testosterone normal/slightly higher range, LH elevated, and FSH and inhibin normal. Excess testosterone produced is peripherally aromatized to estrogen which, together with LH-induced direct secretion of testicular estrogen, results in serum estradiol concentrations higher than those noted in men and boys but lower than those reported in women without CAIS. 1

AIS has been seen to have an increased risk of testicular tumors. Rarely, they present in the prepubertal age group. Studies have suggested an increased tumor risk of greater than 30% in late adulthood if gonadectomy is not done. 7 As the case in the present study was 60 years of age, there was a high chance of testicular tumor. Germ cell tumors are most commonly seen in these patients. Few cases of tumors of nongerminal origin such as Leydig cell tumor, Sertoli cell tumor, sarcoma, and lymphoma have been reported. 8 Present case showed Leydig cell hyperplasia of bilateral gonads; however, there was no evidence of malignancy. Testicular neoplasia is avoided by gonadectomy in such cases once puberty is attained.

Phyllodes tumors are rare primary breast malignancies which are commonly seen between the ages of 40 to 50 years and are mostly seen in female individuals. Few cases of phyllodes in males have been reported with those who have gynecomastia. 3 There have been no reports in the past of AIS with phyllodes. With this being the first reported case of phyllodes in a case of AIS, it is safe to consider the finding of phyllodes in the present case to be due to the presence of bilateral breast development.

Phyllodes present clinically with a breast lump with irregular borders and rapid progression. Radiological examination can be done with USG and mammography. There is no clear indicator of malignancy observed on either USG or mammography as they have features similar to fibroadenoma on mammography and ultrasonography. Although MRI is considered to be extremely sensitive for the detection of breast cancer, it is still difficult to differentiate phyllodes from other breast tumor types. 3 Hence, the mainstay diagnosis is by histopathological examination of a biopsy sample which was done through core needle biopsy in the present study.

Phyllodes are subclassified histologically as benign, borderline, or malignant. 9 The grading is based on the evaluation of criteria such as the stromal component: nuclear pleomorphism, mitotic rate, overgrowth, cellularity, and aspects of tumor margins. In the present study, the histology of the right breast tumor showed exaggerated intracanalicular growth pattern with leaf-like projections extending into dilated lumina with an intact basement membrane. There was also no evidence of abnormal mitosis, ductal carcinoma in situ, apocrine metaplasia, necrosis, or malignancy in the tissue. Hence, the right breast tissue was labeled as benign phyllodes. The left breast mass tissue in the present study showed spindle cells arranged in various patterns with areas of calcification, necrosis, and hyalinization. The tumor also showed infiltrating borders with heterogeneous differentiation along with 10 to 12 mitotic figures/high-power field. These features suggested the histological diagnosis of malignant phyllodes.

Surgery is the mainstay treatment for phyllodes. Breast conservation surgery is attempted with a wide local excision of the tumor to prevent any local recurrences. The margin of resection of the tumor has been arguable but recent studies show that there is no direct relationship between local recurrence rate and the width of negative margins. 10 11 After consulting with the previous studies a decision was taken to do a wide local excision with breast conservation surgery for the right breast. Due to the aggressive nature and extent of the left breast tumor with the malignant grading, the decision for a MRM was done. Wound closure was done with simple polyamide sutures bilaterally with drains for adequate approximation and to prevent seroma accumulation.

Adjuvant radiotherapy, although controversial, has proven to give positive results in various studies. Adjuvant chemotherapy, however, lacks the evidence of providing benefits in reducing local recurrences or improvement in disease-free or overall survival or death. Endocrine therapy has not been shown to be effective in phyllodes as well. This led to the decision of further oncological assessment and the initiation of adjuvant radiotherapy in this study. Follow-up of 9 months showed her to be disease and symptom free.

## Conclusion

CAIS is a syndrome of gonadal dysgenesis which can present with gonadal herniation in the inguinal region. Gonads in CAIS could present uncommonly with conditions such as Leydig cell hyperplasia as in the present case. Due to the presence of breast development, there is a possibility of the development of breast tumors such as phyllodes which are not usually seen in genotypic males. After adequate evaluation and planning, excision of the tumor can be done safely in these patients. Regular follow-up would be mandatory to look out for possible recurrences as is done in female patients with phyllodes tumor. Overall survival can be improved with strategic treatment planning, surgery, and postoperative surveillance.
